# Simulation shows that HLA-matched stem cell donors can remain unidentified in donor searches

**DOI:** 10.1038/srep21149

**Published:** 2016-02-15

**Authors:** Jürgen Sauter, Ute V. Solloch, Anette S. Giani, Jan A. Hofmann, Alexander H. Schmidt

**Affiliations:** 1 DKMS German Bone Marrow Donor Center, Kressbach 1, 72070 Tuebingen, Germany

## Abstract

The heterogeneous nature of HLA information in real-life stem cell donor registries may hamper unrelated donor searches. It is even possible that fully HLA-matched donors with incomplete HLA information are not identified. In our simulation study, we estimated the probability of these unnecessarily failed donor searches. For that purpose, we carried out donor searches in several virtual donor registries. The registries differed by size, composition with respect to HLA typing levels, and genetic diversity. When up to three virtual HLA typing requests were allowed within donor searches, the share of unnecessarily failed donor searches ranged from 1.19% to 4.13%, thus indicating that non-identification of completely HLA-matched stem cell donors is a problem of practical relevance. The following donor registry characteristics were positively correlated with the share of unnecessarily failed donor searches: large registry size, high genetic diversity, and, most strongly correlated, large fraction of registered donors with incomplete HLA typing. Increasing the number of virtual HLA typing requests within donor searches up to ten had a smaller effect. It follows that the problem of donor non-identification can be substantially reduced by complete high-resolution HLA typing of potential donors.

Unrelated hematopoietic stem cell transplantation is a curative therapy for leukemia and other severe blood diseases. Yet, even though more than 26 million potential stem cell donors are currently registered worldwide^1^, finding a donor whose HLA profile matches a patient’s profile is not always straightforward. Most prominently, unrelated donor search is challenged by heterogeneous donor HLA profiles that are present in donor registries. HLA profiles may differ by typing resolution, typing methods applied, and the number of loci typed. These differences result from the growth of the international donor registry over several decades, limited funds for donor HLA typing and different typing strategies of national registries and donor centers.

The current standard for donor-recipient matching includes high-resolution matching of the HLA loci A, B, C and DRB1^2^,^3^,^4^. The DQB1 locus is often also considered in the donor search process and seems to be of relevance, too^5^,^6^. Throughout this work, we regard high-resolution five-locus (HLA-A, -B, -C, -DRB1, -DQB1) donor-recipient matches as complete HLA matches.

In contrast, many HLA profiles of potential donors are still characterized by low resolution HLA information of only few loci. This greatly impedes immediate identification of matching candidates. Therefore, if no fully typed and completely HLA-matched donor is readily identifiable in the registry, a search coordinator may request additional HLA typing of partially typed and potentially HLA-matched donors. Such patient-related HLA typing requests face at least two restrictions: First, the collection of additional HLA typing results may be time-consuming and thus not be suited for very urgent stem cell donor searches. In these cases, it can be more appropriate to select a mismatched unrelated donor or to consider a cord blood or mismatched related (haploidentical) transplantation. Second, budget restrictions normally limit the number of patient-related HLA typing requests. As a consequence, fully HLA-matched donors with incomplete HLA information can remain unidentified during real-life donor searches. We refer to these donors as “hidden donors”. The non-identification of the only or all fully HLA-matched donor/s for a specific patient is an extremely undesired donor search outcome as it may prevent the patient from receiving the preferred therapy or may unnecessarily lead to the selection of a mismatched donor for unrelated transplantation, resulting in lower transplant success rates^2^,^5^.

There is little doubt that such undesired search outcomes really occur in practice. However, it has not been investigated so far if this problem exists only in very few anomalous cases or if all HLA-matched donors remain hidden for a substantial number of patients. In a simulation study, we quantified the share of patients whose donor searches remained unsuccessful despite there was at least one HLA-matched donor in the respective registry. Throughout this work we refer to these searches as “unnecessarily failed searches” (UFS).

## Methods

### Virtual reference registry

We created several virtual donor registries to quantify the share of UFS. One of these registries was designed to mimic the donor file of DKMS Germany as of May 2012 with respect to number of registered donors, share of respective donor typing profiles and donor haplotype frequencies. This very registry served as reference registry throughout this study. Specifically, the reference registry consisted of 2,600,000 virtual donors. Virtual donors’ high-resolution haplotype frequencies followed those of the German population. To estimate five-locus (HLA-A, -B, -C, -DRB1, -DQB1) German haplotype frequencies, we analyzed a large sample of *n* = 370,856 registered stem cell donors with self-reported German ancestry using a previously described implementation^7^ of the expectation-maximization (EM) algorithm^8^.

Due to the large sample used for determination of the haplotype frequency distribution, testing for deviation from HWE could be expected to show significant results^9^. Therefore, the deviation from HWE was assessed using the effect size statistic *W*_*n*_^10^ and the similarity of observed and expected homozygosity rates^11^. The effect size statistic *W*_*n*_ ranges from 0 to 1 where values close to 1 reflect strong disequilibria. As the initial typing resolution was not homogeneous, all typing results were converted to broad serological antigens. Analyses were carried out for each of the five loci individually.

Based on the estimated haplotypes frequencies, donors’ phenotypes were computed assuming random mating. We further assigned one of five HLA typing profiles to each virtual donor. These profiles ranged from “5 HLA loci typed at high resolution” (profile #1) to “HLA-A and -B typed at low resolution” (profile #5). Frequencies of the typing profiles are given in column #8 of Table 1. If profiles #2–#5 were assigned to a donor, the donor’s HLA typing results were obscured accordingly.

### Donor searches

As for virtual donors, we generated 10,000 virtual patients using the same haplotype frequencies as for the donor pool. In average, 99.5% of the virtual patients’ phenotypes contain only common and well-defined (CWD) alleles^12^, while 54 patients have one allele not included in the CWD list. We then simulated virtual donor searches for each virtual patient in order to find fully (10/10) HLA-matched donors within a virtual registry.

If an HLA-matched donor with profile #1 existed for a patient within a registry no further search had to be carried out. Without such HLA-matched profile #1 donors, we simulated virtual HLA typing requests for incompletely typed and potentially HLA-matched donors. Donors were selected for virtual HLA typing in order to maximize virtual patients’ probabilities to find full HLA matches. To do so, matching probabilities (MP) were estimated from the amount of HLA information that corresponded to each donor’s typing level by using the Hap-E Search software^13^. This software uses population-specific haplotype frequencies to estimate the probability that an individual with incomplete HLA information has a specific HLA phenotype. If several donors had identical MP, donors for virtual HLA typing were selected at random. There were no MP thresholds in the donor selection process, i.e., virtual donors with very low MP could also be requested for HLA typing if the applicable typing strategy allowed further typing requests and no donors with higher MP were in the virtual registry.

As a result of a virtual HLA typing, donors’ hidden HLA information was uncovered, thus indicating if the requested donor was a full HLA match for the patient. If within a predefined number of virtual typing requests no HLA-matched donor was found, the virtual donor search was considered unsuccessful. Possible outcomes of donor searches included: An HLA-matched donor was identified; there was no HLA-matched donor in the virtual registry; and there was at least one HLA-matched donor in the virtual registry but all HLA-matched donors remained unidentified in the donor search process. The latter case indicates an unnecessarily failed search (UFS).

Given the relatively low matching probability of donors with typing profile #5 (donors for which only HLA-A and HLA-B have been typed at low resolution), it is a question of considerable practical relevance whether these donors should be selected for additional HLA typing during donor searches. To assess the effect of non-consideration of these donors on the UFS share, we examined two different typing strategies: Typing requests for donors with profile #5 were either allowed (Strategy *A*) or avoided (Strategy *B*).

### Additional virtual registries

To assess the influence of the registry composition on donor search success, we created in total eight additional virtual registries besides the reference registry by modifying only registry size, relative share of HLA profiles, and haplotype frequency (HF) distribution, respectively. Each of these factors was modified separately as follows, while all other parameters of the reference registry remained unchanged (e.g., the virtual registries with different sizes, feature the same relative share of HLA profiles as the reference registry). Regarding registry size, we created four additional registries with *n* = 600,000, *n* = 1,600,000, *n* = 3,600,000 and *n* = 4,600,000 donors, respectively.

Besides, we created two additional registries which varied in terms of HLA profile distribution from the reference registry. In a registry with low data quality the share of high-resolution profiles (i.e., typing profiles #1 and #2) was decreased by 15% compared to the reference registry while in a registry with especially high data quality the respective share was increased by the same amount (see Table 1).

Finally, we modulated the HF distribution from which donor and patient phenotypes were generated. Specifically, we created two additional virtual registries that were characterized either by a higher or lower haplotype diversity. To do so, first the slope of the HF distribution was made shallower for a more diverse HF distribution than in the reference registry and steeper for a less diverse HF distribution. The slope of the distribution was chosen in a way that the cumulated frequencies of the 50 most common haplotypes approximately corresponded to the respective maximum and minimum values obtained in a study focused on ethnic diversity donors in Germany^14^. In that study, donors stemming from Turkey and the United Kingdom had the most and least diverse HF distributions, respectively. Second, HF were normalized to ensure that the sum of all HF was 1. Populations generated from these HF distributions under the assumption of random mating satisfied Hardy-Weinberg equilibrium (HWE) by construction.

As we did not want to focus on effects resulting from different donor and patient populations^15^ in this work, we changed the patient populations according to the donor populations.

For these additional registries the same simulations were carried out as for the reference registry. To even out the influence of chance and reliably estimate donor search success rates, we carried out all simulations including generation of the virtual registries (and patient populations) and conduction of donor searches three times.

## Results

### Haplotype frequencies

The 20 most frequent five-locus (HLA-A, -B, -C, -DRB1, -DQB1) haplotypes with a cumulated frequency of 25.1% are given in Table 2. Results are in good accordance with previously published HF of the German population^16^. A cumulated haplotype frequency of 50% (75%) was reached with the 207 (1,255) most frequent haplotypes. There were 33,405 haplotypes with a frequency larger than 2.7*10^−6^ which was the resolution limit given by 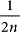 where n = 370,856 was the sample size. These 33,405 haplotypes had a cumulated frequency of 99.998607%. Haplotypes with a lower frequency than the resolution limit are typically regarded as artifacts of the EM algorithm^9^. A full list of all estimated HF is given in the Supplementary Information.

The results regarding the relevance of deviations from HWE are shown in Table 3. For all five HLA loci analyzed, we found relatively small values of *W*_*n*_ between 0.0079 (HLA-A) and 0.0528 (HLA-DQB1). Further, we observed only small deviations from expected homozygosities. The largest excess homozygosity was observed for HLA-C (observed homozygosity 0.1559, expected homozygosity 0.1518, Δ = 0.0041). The only excess heterozygosity occurred in HLA-DQB1 (observed homozygosity 0.1907, expected homozygosity 0.1896, Δ = −0.0011). The results indicate that no relevant deviations from HWE exist^17^.

### Donor search success: Reference registry

**Figure 1:**
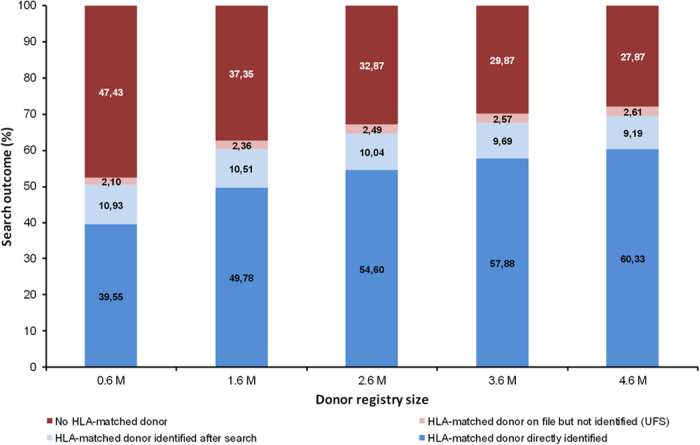
Effect of donor registry size on donor search outcome. The center column (2.6 M donors) shows results for the reference registry. All registries had the same typing profile composition and haplotype frequency distribution as the reference registry. Other parameters: Three typing requests per search were possible, Strategy *A* was applied.

The third column of Fig. 1 shows donor search results for the reference registry when three HLA typing requests were possible and Strategy *A* was applied. For 67.1% of the patients there was at least one completely HLA-matched donor in the virtual donor registry. HLA-matched donors were readily identified for 54.6% of all patients as they had the typing profile #1. For the remaining 45.4% of the patients, virtual typing requests were carried out. Results of these typing requests were as follows: For 32.9% of the patients, virtual typing requests had no success as there was no HLA-matched donor in the reference registry at all. For 10.0% of the patients an HLA-matched donor was found after at most three additional typing requests. Most importantly, for 2.49% of the patients no matching donor was identified after typing of the three most promising potential matches although at least one HLA-matched donor was included in the reference registry.

Now we considered only those patients for whom the search success depended on the activities of the search coordinator, i.e., we excluded patients with readily identifiable profile #1 donors (54.6% of all cases) and patients without HLA-matched donor in the registry (32.9% of all cases). Only 12.5% of all patients remained. Interestingly, for 19.9% of these patients the donor searches failed unnecessarily.

Simulation repeats showed the results were stable. For example, the fraction of patients with UFS was 2.49% ± 0.05% (mean ± standard deviation).

### Effect of search strategy and number of HLA typing requests

**Figure 2:**
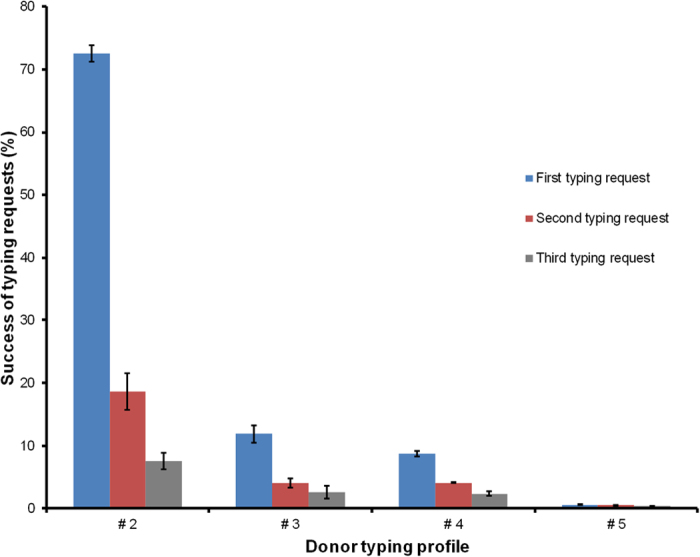
Success of typing requests with respect to donor typing profile. Simulations were carried out using the reference registry. Error bars indicate standard deviations.

Figure 2 shows the success rates of the first three HLA typing requests. Patients with at least one HLA-matched profile #1 donor were not included in the analysis. For potential donors with HLA typing profile #2 the success rate decreased from 72.5% when they were the virtual search coordinator’s first choice to 7.6% when they were requested as third potential donor. As expected, success rates for donors with poorer typing profiles were considerably lower. For donors with typing profile #5 the success rate ranged from 0.60% (first typing request) to 0.34% (third typing request).

The large differences between the success rates of the first and subsequent typing requests seem unusual at first sight. They are partly induced by the fact that virtual donor searches were stopped when one HLA-matched donor had been identified. As a consequence, there often were no second and third typing requests in “easy” donor searches where success rates of typing requests were generally high.

**Figure 3:**
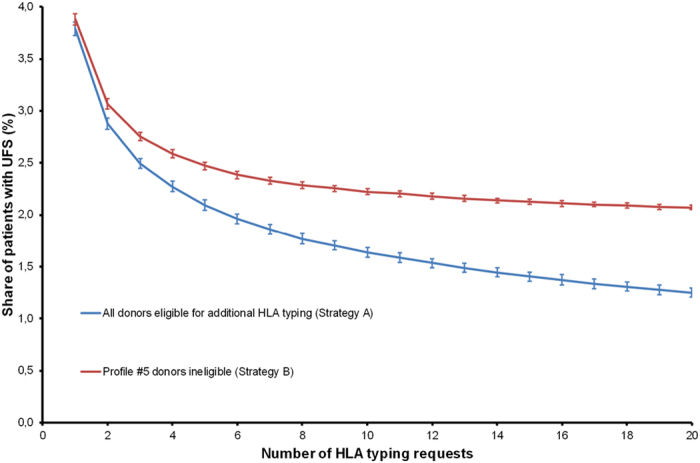
Effect of donor search strategies and number of HLA typing requests. Simulations were carried out using the reference registry. Error bars indicate standard deviations.

Due to the low success rates of typing requests for profile #5 donors, one might argue that these donors should not be requested at all, i.e., that strategy *B* was favorable. The effect of strategies *A* and *B* as well as of the maximum number of HLA typing requests is shown in Fig. 3. Naturally, the share of UFS decreased when more HLA typing requests were possible. With 10 typings requests and Strategy *A*, for example, UFS occurred for 1.64% of all patients compared to 2.49% of all patients when only three requests were allowed and the same strategy was applied.

When donors with typing profile #5 were excluded from HLA typing requests (Strategy *B*), the share of patients with UFS increased. While this increase of UFS was relatively small if only few HLA typing requests were possible (for example, 2.75% with Strategy *B* compared to 2.49% with Strategy *A* when three HLA typing requests were allowed; Δ = 0.26%) it became more pronounced when virtual typing of more potential donors was allowed (for example, 2.07% with Strategy *B* compared to 1.25% with Strategy *A* when 20 HLA typing requests were allowed; Δ = 0.82%). The shape of the curve for Strategy *B* in Fig. 3 suggests that no further significant reduction of the share of patients with UFS can be expected from an ever-growing number of additional HLA typing requests. It has to be noticed that Strategy *B* effectively reduces the size of the reference registry by the number of donors with profile #5.

### Effect of virtual registry size

As expected^18^,^19^, registry size considerably influenced the share of patients for which at least one HLA-matched donor was included in the virtual registry. The respective fraction of patients ranged from 52.6% (0.6 M donors) to 72.1% (4.6 M donors) (Fig. 1). Similarly, also the share of patients for whom an HLA-matched donor was readily identified without further HLA typing requests increased substantially from 39.5% (0.6 M donors) to 60.3% (4.6 M donors). The percentages of the other patient groups showed lower variability: The share of patients with HLA-matched donors identified within the search decreased slightly from 10.9% (0.6M donors) to 9.2% (4.6 M donors) while the fraction of patients with UFS increased from 2.10% (0.6M donors) to 2.61% (4.6 M donors).

Again, we also considered only those patients for whom the search success depended on the activities of the search coordinator. Among these patients, the fraction of patients with UFS increased from 16.1% (0.6 M donors) to 22.2% (4.6 M donors). Summarized, these results show that additional donor recruitment alone is not suited to resolve the UFS problem.

### Effect of typing profile composition

**Figure 4:**
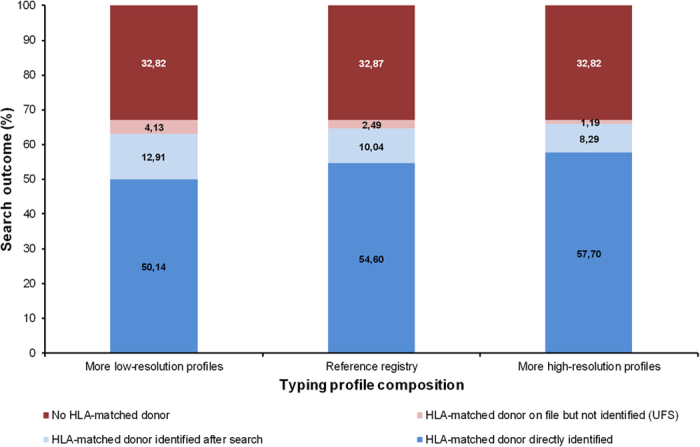
Effect of typing profile distribution on donor search outcome. For details regarding the registries with more high-resolution and low-resolution profiles see Methods section. All registries included 2.6 M donors and had the same haplotype frequency distribution as the reference registry. Other parameters: Three typing requests per search were possible, Strategy *A* was applied.

Variation of the registry composition with respect to typing profiles had the most pronounced effect on the share of patients with UFS (Fig. 4). This share was more than three times higher in the “low-resolution registry” (4.13%) than in the “high-resolution registry” (1.19%).

When only patients for whom the search success depended on the activities of the search coordinator were considered, the share of patients with UFS ranged from 12.5% (high-resolution registry) to 24.2% (low-resolution registry). These observations show that it is possible to considerably reduce the problem of non-identification of HLA-matched donors by more complete HLA typing.

In doing so, the share of patients with readily identifiable HLA-matched donors also increased from 50.1% (low-resolution registry) to 57.7% (high-resolution registry). In practice, this higher share of donors with readily identifiable donors should reduce donor search times.

### Effect of haplotype frequency distribution

**Figure 5:**
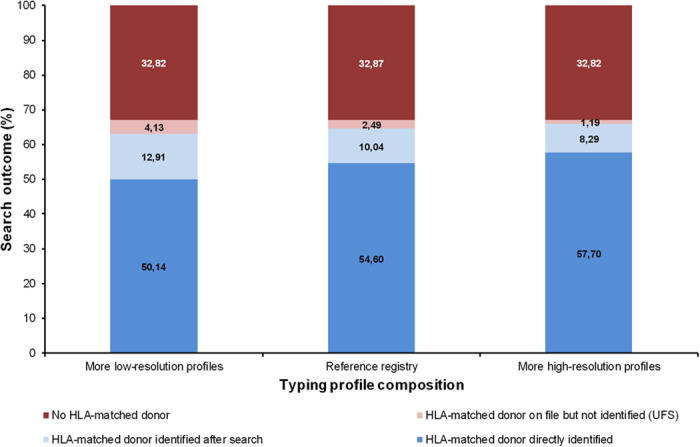
Effect of haplotype frequency distribution of donor and patient populations on donor search outcome. For details regarding the populations with more diverse and less diverse haplotype frequency distributions see Methods section. All registries included 2.6 M donors and had the same typing profile composition as the reference registry. Other parameters: Three typing requests per search were possible, Strategy *A* was applied.

Unsurprisingly^14^,^15^, genetic diversity of donor and patient populations had a strong impact on the share of patients with no HLA-matched donor in the virtual registry. The respective fraction of patients was 22.8% for low genetic diversity and 44.1% for high genetic diversity (Fig. 5). The fraction of patients with at least one directly identifiable HLA-matched donor also differed considerably between the various scenarios and went from 41.8% (high diversity) to 67.2% (low diversity). The share of patients for whom HLA-matched donors were identified within the search ranged from 8.1% (low diversity) to 11.1% (high diversity), while the share of patients with UFS varied from 1.85% (low diversity) to 2.94% (high diversity).

When only those searches where success depended on the search coordinator’s activities were taken into account, the share of patients with UFS varied only slightly and ranged from 18.6% (low diversity) to 20.9% (high diversity). Taken together, these results show that the problem of non-identification of HLA-matched donors is slightly more prominent for donor and patient populations with high genetic diversity.

## Discussion

Unrelated donor searches are hampered by heterogeneous HLA profiles of registered stem cell donors. Most prominently, incomplete donor HLA typing (low-resolution typing and/or missing loci) impedes fast and safe identification of completely matched donors. To quantify these challenges to donor search, we carried out simulations based on haplotype frequencies of the German population using simplifying assumptions regarding the donor search process. The simulation approach is appropriate to analyze the probability of non-identification of registered stem cell donors who are full HLA matches to patients in need of a transplant. This question could otherwise hardly be answered without extensive typing of so far incompletely typed donors.

The main results of our study are as follows: First, the non-identification of fully HLA-matched donors is a problem of practical relevance and not limited to only very few cases. This result is consistent with observations we made in a qualitative practical study^20^. Second, more complete HLA typing of registered stem cell donors substantially reduces the problem of donor non-identification. Third, compared to completeness of HLA typing information, size and genetic composition of a donor registry have less impact on the fraction of patients with unnecessarily failed donor searches. Fourth, the increase of the number of HLA typing requests that are allowed within a donor search reduces the fraction of UFS only moderately. This holds especially when poorly HLA-typed donors (profile #5 donors in our study) are not eligible for additional HLA typing.

There are several limitations to our analysis. First, our virtual registries showed some simplifications compared to real-life registries. They included, for example, only donors with 5 different typing levels. In reality, most registries include considerably more various typing levels. Of greater relevance is probably the fact that we neglected the existence of donors with “intermediate” typing resolution. In practice, these typing results often originated from SSO typing. The quality of typing results with intermediate resolution is sometimes close to that of high-resolution typing. In other cases, however, it is hardly superior to low-resolution typing. Therefore, the modeling of intermediate typing would have caused considerable methodological difficulties. Besides, we did not consider the non-availability of registered donors or the existence of additional registries with different genetic characteristics that can be included in the donor search in practice.

Second, we also used a simplified search process in our model. Real search coordinators might have different approaches to request donors for additional HLA typing that could be more or less efficient than strictly following the calculated matching probabilities. Besides, our assumption that the maximum number of typing requests is identical for all patients is probably not very realistic. In practice, many search coordinators rather administer a defined budget for certain numbers of donor searches and thus are able to “save” typing requests from “easy” searches for more complicated searches.

Third, search success was in our model defined in a simpler way than in practice. We regarded only the identification of 10/10 matched donors as search success, thus neglecting the fact that transplant physicians often accept donors with a single mismatch. Besides, we did not consider non-HLA factors with potential relevance for the donor search as, for example, age, gender, blood groups or infectious disease markers^5^,^21^,^22^,^23^. Besides, our model did not include time as a dimension. In practice, a donor search may fail because the patient’s health condition deteriorates during the search and does not allow transplantation anymore. The non-consideration of time effects reduces the benefits of complete donor typing in our model as the reduction of search times that can be expected from complete typing in practice is neglected.

Taken together, the limitations of the model simulations suggest that our quantitative results have to be interpreted carefully. It is, however, not plausible that our main results as described above are not valid. Therefore, the strong effect of donor typing levels on the occurrence of the hidden donor problem as shown by our simulation results encourages investments in typing quality of potential stem cell donors. This recommendation refers both to new registrants and to donors who are already on file with so far incomplete HLA information. Complete HLA typing of potential stem cell donors had also been shown to be advantageous for operations and planning of donor centers and registries before^15^,^24^. In the past, cost considerations often led to incomplete typing (not all loci typed and/or several loci typed only at low or intermediate resolution). In the current era of HLA typing by next-generation sequencing and related cost reductions^25^,^26^, it is desirable that more and more donor centers and registries will carry out complete HLA typing for all new donors and for selected donors who are already on the file.

Analyses of donor file size-dependent probabilities to find HLA-matched unrelated stem cell donors have been published for several populations and various matching levels^27^,^28^,^29^,^30^. These results provide valuable insights for strategic donor registry planning. However, the underlying calculations neglect the problem of donor non-identification and thus overestimate matching probabilities as they occur in practice.

Summarized, our simulation study demonstrated that completely HLA-matched donors were not necessarily identified during donor searches. In our framework, this hidden donor problem occurred for about 1–4% of donor searches. It slightly scaled with donor registry size and showed weak dependencies from the haplotype frequency distribution. However, the share of unnecessarily failed donor searches decreased substantially with increasing number of donors with complete high-resolution HLA typing profiles in the registry. These results underline the need for complete HLA typing of potential stem cell donors.

## Additional Information

**How to cite this article**: Sauter, J. *et al.* Simulation shows that HLA-matched stem cell donors can remain unidentified in donor searches. *Sci. Rep.* **6**, 21149; doi: 10.1038/srep21149 (2016).

## Supplementary Material

Supplementary Information

## Figures and Tables

**Table 1:** Composition of virtual registries with respect to HLA typing profiles.

Donor typing profile	HLA locus					Typing profiles (%)		
	A	B	C	DRB1	DQB1	Low data quality registry	Reference registry	High data quality registry
#1	H	H	H	H	H	25	30	35
#2	H	H	H	—	—	18	28	38
#3	L	L	—	H	—	23	18	13
#4	L	L	—	—	—	27	17	7
#5	L	L	—	—	—	7	7	7
Total						100	100	100

H = High-resolution typing, L = Low-resolution typing, –  =  not typed at all. Example: 23% of the donors of the low data quality registry have typing profile #3.

**Table 2:** Top 20 haplotypes estimated from German donors (sample size: *n* = 370,856).

Rank	Haplotype (HLA-A* ~ B* ~ C* ~ DRB1* ~ DQB1*)	Frequency (%)	Cumulated Frequency (%)
1	01:01g^‡^ ~ 08:01g ~ 07:01g ~ 03:01 ~ 02:01g	6.06	6.06
2	03:01g ~ 07:02g ~ 07:02g ~ 15:01 ~ 06:02	3.31	9.37
3	02:01g ~ 07:02g ~ 07:02g ~ 15:01 ~ 06:02	1.94	11.30
4	03:01g ~ 35:01g ~ 04:01g ~ 01:01 ~ 05:01	1.58	12.88
5	02:01g ~ 15:01g ~ 03:04g ~ 04:01 ~ 03:02g	1.24	14.12
6	02:01g ~ 44:02g ~ 05:01g ~ 04:01 ~ 03:01g	1.06	15.18
7	29:02g ~ 44:03 ~ 16:01 ~ 07:01 ~ 02:01g	1.03	16.21
8	02:01g ~ 40:01g ~ 03:04g ~ 13:02 ~ 06:04g	1.02	17.23
9	02:01g ~ 13:02g ~ 06:02g ~ 07:01 ~ 02:01g	0.84	18.07
10	01:01g ~ 57:01g ~ 06:02g ~ 07:01 ~ 03:03g	0.81	18.89
11	23:01g ~ 44:03g ~ 04:01g ~ 07:01 ~ 02:01g	0.74	19.62
12	02:01g ~ 57:01g ~ 06:02g ~ 07:01 ~ 03:03g	0.73	20.35
13	24:02g ~ 07:02g ~ 07:02g ~ 15:01 ~ 06:02	0.72	21.07
14	30:01g ~ 13:02g ~ 06:02g ~ 07:01 ~ 02:01g	0.71	21.78
15	02:01g ~ 15:01g ~ 03:03g ~ 13:01g ~ 06:03g	0.66	22.44
16	11:01g ~ 35:01g ~ 04:01g ~ 01:01 ~ 05:01	0.59	23.03
17	02:01g ~ 08:01g ~ 07:01g ~ 03:01 ~ 02:01g	0.58	23.61
18	25:01g ~ 18:01g ~ 12:03g ~ 15:01 ~ 06:02	0.55	24.16
19	01:01g ~ 07:02g ~ 07:02g ~ 15:01 ~ 06:02	0.50	24.66
20	02:01g ~ 44:02g ~ 05:01g ~ 13:01g ~ 06:03g	0.44	25.10

^‡^g groups were defined as described before^16^.

**Table 3:** Effect size statistics and observed and expected homozygosities.

Locus	Effect size statistics *W*_*n*_	Observed homozygosity	Expected homozygosity
HLA-A*	0.0079	0.1563	0.1554
HLA-B*	0.0208	0.0798	0.0761
HLA-C*	0.0239	0.1559	0.1518
HLA-DRB1*	0.0202	0.1122	0.1136
HLA-DQB1*	0.0528	0.1907	0.1896

## References

[b1] BMDW. http://bmdw.org/, < http://bmdw.org/> (accessed on March 27th, 2015).

[b2] Lee S. J. *et al.* High-resolution donor-recipient HLA matching contributes to the success of unrelated donor marrow transplantation. Blood 110, 4576–4583, doi: 10.1182/blood-2007-06-097386 (2007). 17785583

[b3] Bray R. A. *et al.* National marrow donor program HLA matching guidelines for unrelated adult donor hematopoietic cell transplants. Biology of blood and marrow transplantation: journal of the American Society for Blood and Marrow Transplantation 14, 45–53, doi: 10.1016/j.bbmt.2008.06.014 (2008). 18721780

[b4] Shaw B. E., Arguello R., Garcia-Sepulveda C. A. & Madrigal J. A. The impact of HLA genotyping on survival following unrelated donor haematopoietic stem cell transplantation. British journal of haematology 150, 251–258, doi: 10.1111/j.1365-2141.2010.08224.x (2010). 20560963

[b5] Fürst D. *et al.* High-resolution HLA matching in hematopoietic stem cell transplantation: a retrospective collaborative analysis. Blood 122, 3220–3229, doi: 10.1182/blood-2013-02-482547 (2013). 24046013

[b6] Fernandez-Vina M. A. *et al.* Multiple mismatches at the low expression HLA loci DP, DQ, and DRB3/4/5 associate with adverse outcomes in hematopoietic stem cell transplantation. Blood 121, 4603–4610, doi: 10.1182/blood-2013-02-481945 (2013). 23596045 PMC3668493

[b7] Schmidt A. H. *et al.* High-resolution human leukocyte antigen allele and haplotype frequencies of the Polish population based on 20,653 stem cell donors. Human immunology 72, 558–565, doi: 10.1016/j.humimm.2011.03.010 (2011). 21513754

[b8] Excoffier L. & Slatkin M. Maximum-likelihood estimation of molecular haplotype frequencies in a diploid population. Mol Biol Evol 12, 921–927 (1995). 7476138 10.1093/oxfordjournals.molbev.a040269

[b9] Eberhard H. P. *et al.* Estimating unbiased haplotype frequencies from stem cell donor samples typed at heterogeneous resolutions: a practical study based on over 1 million German donors. Tissue antigens 76, 352–361, doi: 10.1111/j.1399-0039.2010.01518.x (2010). 20604895

[b10] Klitz W., Stephens J. C., Grote M. & Carrington M. Discordant patterns of linkage disequilibrium of the peptide-transporter loci within the HLA class II region. American journal of human genetics 57, 1436–1444 (1995). 8533774 PMC1801434

[b11] Fallin D. & Schork N. J. Accuracy of haplotype frequency estimation for biallelic loci, via the expectation-maximization algorithm for unphased diploid genotype data. American journal of human genetics 67, 947–959, doi: 10.1086/303069 (2000). 10954684 PMC1287896

[b12] Mack S. J. *et al.* Common and well-documented HLA alleles: 2012 update to the CWD catalogue. Tissue antigens 81, 194–203, doi: 10.1111/tan.12093 (2013). 23510415 PMC3634360

[b13] Hofmann J. *et al.* How to plant a haplotype tree: speeding up haplotype frequency-based searches. Tissue antigens 77, 409 (2011).

[b14] Pingel J. *et al.* High-resolution HLA haplotype frequencies of stem cell donors in Germany with foreign parentage: how can they be used to improve unrelated donor searches? Human immunology 74, 330–340, doi: 10.1016/j.humimm.2012.10.029 (2013). 23200758

[b15] Schmidt A. H., Sauter J., Pingel J. & Ehninger G. Toward an optimal global stem cell donor recruitment strategy. PloS one 9, e86605, doi: 10.1371/journal.pone.0086605 (2014). 24497958 PMC3907384

[b16] Schmidt A. H. *et al.* Estimation of high-resolution HLA-A, -B, -C, -DRB1 allele and haplotype frequencies based on 8862 German stem cell donors and implications for strategic donor registry planning. Human immunology 70, 895–902, doi: 10.1016/j.humimm.2009.08.006 (2009). 19683023

[b17] Maiers M., Gragert L. & Klitz W. High-resolution HLA alleles and haplotypes in the United States population. Human immunology 68, 779–788, doi: 10.1016/j.humimm.2007.04.005 (2007). 17869653

[b18] Dehn J. *et al.* 8/8 and 10/10 high-resolution match rate for the be the match unrelated donor registry. Biology of blood and marrow transplantation: journal of the American Society for Blood and Marrow Transplantation 21, 137–141, doi: 10.1016/j.bbmt.2014.10.002 (2015). 25307419

[b19] Bergstrom T. C., Garratt R. J. & Sheehan-Connor D. One chance in a million: Altruism and the bone marrow registry. American Economic Review 99, 1309–1334 (2009). 10.1257/aer.99.4.1309 29508972

[b20] Schmidt A. H. *et al.* Support of unrelated stem cell donor searches by donor center-initiated HLA typing of potentially matching donors. PloS one 6, e20268, doi: 10.1371/journal.pone.0020268 (2011). 21625451 PMC3098867

[b21] Ljungman P., Hakki M. & Boeckh M. Cytomegalovirus in hematopoietic stem cell transplant recipients. Hematology/oncology clinics of North America 25, 151–169, doi: 10.1016/j.hoc.2010.11.011 (2011). 21236396 PMC3340426

[b22] Cooley S. *et al.* Donor selection for natural killer cell receptor genes leads to superior survival after unrelated transplantation for acute myelogenous leukemia. Blood 116, 2411–2419, doi: 10.1182/blood-2010-05-283051 (2010). 20581313 PMC2953880

[b23] Blin N. *et al.* Impact of donor-recipient major ABO mismatch on allogeneic transplantation outcome according to stem cell source. Biology of blood and marrow transplantation: journal of the American Society for Blood and Marrow Transplantation 16, 1315–1323, doi: 10.1016/j.bbmt.2010.03.021 (2010). 20353831

[b24] Müller C., Feldman U., Bochtler W., Morsch S. & Schmidt A. The Effect of age, gender and typing resolution on the probability of stem cell donation. Human immunology 73, 121 (2012).

[b25] Lange V. *et al.* Cost-efficient high-throughput HLA typing by MiSeq amplicon sequencing. BMC genomics 15, 63, doi: 10.1186/1471-2164-15-63 (2014). 24460756 PMC3909933

[b26] Ehrenberg P. K. *et al.* High-throughput multiplex HLA genotyping by next-generation sequencing using multi-locus individual tagging. BMC genomics 15, 864, doi: 10.1186/1471-2164-15-864 (2014). 25283548 PMC4196003

[b27] Müller C. R., Ehninger G. & Goldmann S. F. Gene and haplotype frequencies for the loci HLA-A, HLA-B, and HLA-DR based on over 13,000 german blood donors. Human immunology 64, 137–151 (2003). 12507825 10.1016/s0198-8859(02)00706-1

[b28] Kollman C. *et al.* Assessment of optimal size and composition of the U.S. National Registry of hematopoietic stem cell donors. Transplantation 78, 89–95 (2004). 15257044 10.1097/01.tp.0000132327.40702.97

[b29] Schmidt A. H. *et al.* Regional differences in HLA antigen and haplotype frequency distributions in Germany and their relevance to the optimization of hematopoietic stem cell donor recruitment. Tissue antigens 76, 362–379, doi: 10.1111/j.1399-0039.2010.01520.x (2010). 20545902

[b30] Buhler S., Nunes J. M., Nicoloso G., Tiercy J. M. & Sanchez-Mazas A. The heterogeneous HLA genetic makeup of the Swiss population. PloS one 7, e41400, doi: 10.1371/journal.pone.0041400 (2012). 22848484 PMC3405111

